# Study on the Calculation Method of Hansen Solubility Parameters of Fuel Cell Ionomers

**DOI:** 10.3390/polym17070840

**Published:** 2025-03-21

**Authors:** Chao Meng, Shang Li, Qianyun Wu, Mengyu Li, Shenao Tian, Haolin Tang, Mu Pan

**Affiliations:** 1 State Key Laboratory of Advanced Technology for Materials Synthesis and Processing, Wuhan University of Technology, Luoshi Road 122#, Wuhan 430070, China; 331045@whut.edu.cn (C.M.);; 2 National Energy Key Laboratory for New Hydrogen-Ammonia Energy Technologies, Foshan Xianhu Laboratory, No. 1 Yangming Road, Danzao Town, Nanhai District, Foshan 528200, China; 3 Hubei Key Laboratory of Fuel Cells, Wuhan University of Technology, Wuhan 430070, China

**Keywords:** perfluorosulfonic acid ionomer, Nafion, Hansen solubility parameter, inverse gas chromatography

## Abstract

Accurately determining the Hansen solubility parameters (HSPs) of fuel cell ionomers is crucial for optimizing the dispersion and dispersive state of the ionomer in fuel cell catalyst inks. This directly impacts the structure and coating process of the catalyst layer in proton exchange membrane fuel cells (PEMFCs). The Hansen solubility parameters (HSPs) of the Nafion ionomer were calculated by the Hansen solubility parameter software (HSPiP), inverse gas chromatography (IGC), and group contribution methods. The applicability and accuracy of the different algorithms are discussed. It was found that the solubility parameters (SPs) measured by the HSPiP method were higher, while the SPs measured by the IGC and group contribution methods were lower. However, for the ionomer with both a hydrophobic backbone and hydrophilic side chain, the HSPiP method offered a more reasonable HSP determination method. The dual HSPs of Nafion calculated by the HSPiP method were found to be *δ*_d_ = 16.4 MPa^1/2^ (dispersion force), *δ*_p_ = 10.5 MPa^1/2^ (polar interaction), and *δ*_h_ = 8.9 MPa^1/2^ (hydrogen bonding) for the hydrophobic backbone and *δ*_d_ = 15.2 MPa^1/2^, *δ*_p_ = 11.7 MPa^1/2^, and *δ*_h_ = 15.9 MPa^1/2^ for the hydrophilic side chain. These results provide a thermodynamic basis for solvent design in fuel cell catalyst-layer fabrication.

## 1. Introduction

**Figure 1:**
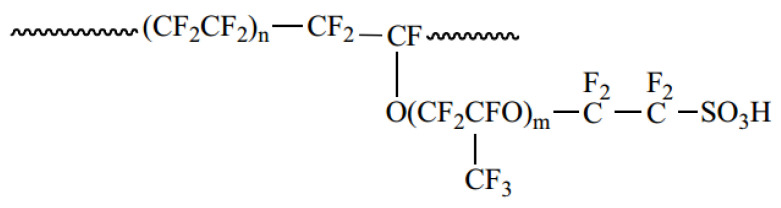
Chemical structure of the Nafion ionomer.

Proton exchange membrane fuel cells (PEMFCs), as a key technology for hydrogen energy power generation, have become a critical pathway for reducing carbon emissions in heavy-duty vehicles and power stations due to their advantages of cleanliness and high energy density [1,2]. To reduce the high hydrogen consumption linked with the high powers and long operating times of heavy-duty applications, it is necessary to further improve the energy conversion efficiency of fuel cells, meaning the working voltages should be above 0.8 V. However, due to electrochemical polarization, the current density decreases with the increase in the operating voltage of the fuel cell [3]. To improve the current density of the fuel cell at 0.8 V, it is necessary to optimize the structure of the catalyst layer and maximize the oxygen-reducing reaction activity of the catalyst. The catalyst layer consists of both the catalyst and ionomer. During the preparation of the catalyst ink for the fuel cell, the interactions between different solvents and the ionomer need to be considered and fine-tuned. These interactions determine the dispersion state of the ionomer [4] and rheological properties of the ink [5,6]. Consequently, they impact the porous structure [7], surface cracks, and overall performance of the fuel cell [8]. Currently, catalyst inks are generally based on a perfluorosulfonic acid ionomer (Nafion^®^). Nafion^®^ is a perfluorosulfonic acid-based polymer developed by DuPont in the late 1960s [9]. It consists of a hydrophobic tetrafluoroethylene backbone and hydrophilic perfluorinated vinyl ether side chains with a sulfonic acid end group (-SO_3_H) [10]. Figure 1 shows the chemical structure of Nafion (where n = 6~13, m ≤ 1). The hydrophobic PTFE backbone in the Nafion structure provides good thermal and chemical stability, and the hydrophilic perfluorinated sulfonic acid side chains provide channels for proton conduction. As a result, Nafion shows high ionic conductivity, as well as mechanical and chemical stability. To optimize the performance of Nafion-based catalyst layers, the selection of an appropriate dispersion solvent is critical, as effective solvents enable optimal ionomer dispersion through enhanced ionomer-solvent molecular interactions.

The solubility parameter (SP) is a key thermodynamic parameter of polymers, which plays an important role in selecting a solvent to dissolve the polymer. The SP was first proposed by Hildebrand and defined as the square root of the cohesive energy density, as shown in Equation (1) [11,12]:

(1) $$\delta_{\mathrm{Hil}}={\left(\frac{E_{\mathrm{coh}}}{V}\right)}^{1/2}=e_{\mathrm{coh}}^{1/2}={\left[\left(\Delta H_{V}-RT\right)/V\right]}^{1/2}$$

where *δ*_Hil_ is the Hildebrand solubility parameter, cal^1/2^ cm^−3/2^ or MPa^1/2^, *E*_coh_ is the cohesive energy, which refers to the energy required for 1 mol of condensates to vaporize in response to intermolecular forces; *e*_coh_ is the cohesive energy density; Δ*H*_V_ is the heat of vaporization; *V* is the molar volume; *R* is the universal gas constant; and *T* is the absolute temperature.

In a study by Yeo in 1980 [13], the dual Hildebrand SPs of Nafion were calculated, one corresponding to the side chain and the other corresponding to the backbone. Since then, this result of Nafion’s SPs has been extensively used by researchers [14,15,16]. However, Hildebrand SPs are inaccurate for systems with higher polarity or hydrogen bonds [17]. To address this, Hansen proposed three-dimensional SPs, also called Hansen solubility parameters (HSPs). As shown in Equation (2), the total SP, *δ*_t_, consists of three components: dispersion (*δ*_d_), polar (*δ*_p_), and hydrogen bond (*δ*_h_) forces. The HSP theory has been widely applied to nanoparticles and pigments [18], organic semiconductors [19], ionic liquids [20], art protection [21], and solid-phase extraction [22].

(2) $$\delta_{t}^{2}=\delta_{d\;}^{2}+\delta_{p\;}^{2}+\delta_{h}^{2}$$

Typically, Hildebrand and Hansen SPs are calculated to determine the cohesive energy of the polymer. The cohesive energy of the polymer depends on the heat of vaporization, which is difficult to measure. Therefore, the HSPs of a polymer can only be measured by indirect methods. The traditional HSPs are measured by static methods, such as the swelling, viscosity, and turbidity methods, but these methods are time-consuming and have relatively large errors. At present, there are three accurate calculation methods used for HSPs: (1) The Hansen solubility parameters in practice (HSPiP) method, which simulates the effect of a series of organic solvents with known properties on the swelling and dissolution of materials using HSP software (Version 5.1.03) invented by Hansen teams; the HSPs of polymers are calculated to guide the solvent selection and intermiscibility [23,24]. (2) Inverse gas chromatography (IGC) is a rapid, direct, and sensitive method to study the interaction between the polymer and solvent. It is widely used to determine SPs of the polymer, lignin, ionic liquid, and other organic materials and structural characteristics [20,25,26]. (3) The group contribution method estimates SPs of polymers and pure organic compounds based on the contributions of their functional groups [18,27,28]. This method is simple and convenient, as it does not require dissolution and swelling experiments and only needs to make a calculation based on the functional groups in the chemical structure of the polymer. Further study is needed to identify which method is more suitable for calculating the HSPs of a PFSA ionomer (Nafion) in fuel cells.

In this work, the HSP computational method for the PFSA ionomer Nafion was studied, and the HSPs of the PFSA ionomer Nafion were calculated using the HSPiP, IGC, and group contribution methods. The applicability and accuracy of the different algorithms are discussed. The determination of a reasonable calculation method for HSPs provides a basis for selecting dispersing solvents for perfluorosulfonic acid ionomers with different side chain lengths in the future.

## 2. Materials and Methods

### 2.1. Materials and Solvents

In this study, the PFSA ionomer Nafion-H (IEC ≤ 0.9 mmol/g, 2–10 μm powder, Zhengzhou Alfa Chemical Co., Ltd., Zhengzhou, China) was used; its chemical structure is shown in Figure 1. The Nafion was oven-dried at 60 °C to remove water and volatile chemicals (VOCs). Fifty different solvents were used in the HSPiP solubility sphere test, purchased from Shanghai Macklin Biochemical Technology Co., Ltd., Shanghai, China. All reagents used in this work were of analytical grade and were used without further purification.

### 2.2. HSPiP Software Method

#### 2.2.1. Experimental Principle of the HSPiP Method

According to the solubility phenomenon, the HSPiP method was used to calculate the HSPs of polymers based on the principle of ‘like dissolves like’. In this work, the HSPiP software developed by Hansen was used to predict the HSPs. Hansen recommends using 20–50 solvents for dissolution and swelling experiments, and the more solvents used, the more accurate the HSPs of the substance. It is assumed that a solubility sphere would be produced on a graph with the axes of *δ*_d_, *δ*_p_, and *δ*_h_ [17]. The HSPiP software can optimize the spherical center coordinate and interaction radius (*R*_o_), such that all good and bad solvents are inside and outside the solubility sphere, respectively. Furthermore, the central coordinate of the solubility sphere is considered the HSPs of the polymer. The distance in the solubility sphere between solvent and polymer, *R*_a_, can be calculated from Equation (3) [17]:

(3) $$R_{a}^{2}=\left[4{\left(\delta_{d\;}^{S}-\delta_{d}^{P}\right)}^{2}+{\;\left(\delta_{p}^{S}-\delta_{p\;}^{P}\right)}^{2}+{\left(\delta_{h}^{S}-\delta_{h}^{P}\right)}^{2}\right]$$

Superscripts *S* and *P* denote the solvent and polymer, respectively. *R*_o_ is the Hansen solubility sphere radius of the polymer simulated using the HSPiP method. If *R*_a_ is smaller than *R*_o_, the solvent can dissolve or strongly swell the polymer. If *R*_a_ is greater than *R*_o_, the solvent does not swell the polymer, and there is no obvious interaction. Another very useful parameter is the relative energy difference (RED), which is given by Equation (4). If there is no energy difference between the solvent and the polymer (RED = 0), the solvent can dissolve the polymer to the maximum extent. If the solvent can dissolve the polymer and is a good solvent (RED < 1), the smaller the RED, the better the dissolution effect. If the solvent is a poor solvent outside the radius of action of the polymer (RED > 1), it shows a poor dissolution effect with the polymer. If the solvent is at the boundary of the SP sphere (RED = 1), it belongs to the boundary solvent and is also the key solvent for determining the HSPs of the polymer [24].

RED = *R*_a_/*R*_o_ (4)

Generally, HSPs for each phase of a material (solid, liquid, and gas) will have single values. Therefore, all materials have a single sphere in the Hansen 3D diagram. However, for some substances, such as ionic liquid and surfactant [23,29], the Hansen 3D diagram is best drawn as two spheres, i.e., hydrophobic and hydrophilic spheres. The dual sphere option (“Double Sphere”) [30] integrated in the HSPiP software can be used to evaluate HSPs for surfactants or ionic liquids. In this work, we applied the dual sphere approach to Nafion polymers.

#### 2.2.2. HSPiP Experiment

Fifty types of pure solvents were used to dissolve and swell the Nafion polymer, and the Hansen solubility sphere of the Nafion polymer was simulated according to the experimental results. To conduct the test, 0.5 g of the Nafion-H powder and 5 mL of the test solvent were taken in a vial, sealed, and immersed in a 25 °C incubation bath. The dissolution and swelling process occurred under magnetic stirring using a PTFE-coated stir bar, purchased from Bikeman Biotechnology Co., Ltd., Changde, China, with the degree of solvent interaction continuously monitored at 2 h intervals until equilibrium was achieved within a 48 h period. The results of the solubility test were determined by visual observation based on a scale from 1 to 6, which was established by Hansen [17]: 1—Soluble, 2—Almost soluble, 3—Strongly swollen, slight solubility, 4—Swollen, 5—Little swelling, and 6—No visible effect. Examples of dissolution and swelling at different scales (scale 1–6) are shown in Figure S1. The solubility test results were directly input into the software, fitting analysis was performed by the “Classic” (single sphere) and “Double Sphere” (double sphere) methods, respectively, and HSPs and solubility spheres of the Nafion polymer were the output.

### 2.3. Inverse Gas Chromatography (IGC) Method

#### 2.3.1. Experimental Principle of the IGC Method

IGC techniques are commonly used to characterize the thermodynamic properties of polymers. In the IGC theory, the specific retention volume $V_{g}^{0}$ is used to characterize the elution behavior of the probe in the column and can be expressed as follows [20]:

(5) $$V_{g}^{0}=\frac{273.15}{mT_{a}}F\frac{P_{o}-P_{w}}{P_{o}}\left(t_{r}-t_{0}\right)\frac{{3\left(P_{i}\;/P_{o}\right)}^{2}-1}{{2\left(P_{i\;}/\;P_{o}\right)}^{3}-1}$$

where *m* is the mass of the polymer stationary phase in the column, *T*_a_ is the column temperature, *F* is the flow rate of the carrier gas measured at the column temperature *T*_a_, *t*_r_ − *t*_0_ is the net retention time of the probe, *P*_w_ is the saturated vapor pressure of water, and *P*_i_ and *P*_o_ are the pressures at the inlet and outlet of the column, respectively.

According to the Flory–Huggins theory, the interaction parameters, $\chi_{12}^{\infty }$, of solvent 1 in stationary phase 2 under infinite dilution can be calculated as follows:

(6) $$\chi_{12}^{\infty }=ln\frac{273.15RV_{2}}{P_{1}^{0}V_{g}^{0}V_{1}}-P_{1}^{0}\frac{B_{11}-V_{1}}{RT}-\;1$$

where *R* is the universal gas constant, *V*_1_ is the molar volume of the solvent probe, *V*_2_ is the molar volume of the polymer in the stationary phase, *T* is the column temperature, and $P_{1}^{0}$ is the saturated vapor pressure of the solvent probe, and can be calculated by Equation (7):

(7) $$lgP_{1}^{0\;}=A-\frac{B}{T\;+C}$$

where *A*, *B*, and *C* are constants, and *B*_11_ is the second virial coefficient of the probe solvent, which can be expressed by Equation (8):

(8) $$\frac{B_{11}}{V_{c}}=0.430-0.886\frac{T_{c}}{T}-0.694(\frac{T_{c}}{T}{)}^{2}-0.0375\left(n-1\right)(\frac{T_{c}}{T}{)}^{4.5}$$

where *n* is the number of carbon atoms in the solvent, *V*_c_ is the critical molar volume of the solvent, and *T*_c_ is the critical temperature of the solvent.

The Hildebrand solubility parameter, *δ*_2_, of the polymer can be calculated according to the following equation [31]:

(9) $$\left(\frac{\delta_{1}^{2}}{RT}-\frac{\chi_{12}^{\infty }}{V_{1}}\right)=\left(\frac{2\delta_{2}}{RT}\right)\delta_{1}-\frac{\delta_{2}^{2}}{\mathrm{RT}}$$

In this expression, *δ*_1_ represents the solubility parameters of the solvents, which can be found in the literature [32]. According to Equation (9), *δ*_2_ is obtained from the straight line with a slope of 2*δ*_2_/*RT*.

Traditional IGC can only calculate the one-dimensional Hildebrand solubility parameter (*δ*_2_) of the material. To make the Hildebrand solubility parameter of the material three-dimensional, Abbott et al. proposed the method of fitting the experimental value ($\chi_{\exp}$) and calculated value ($\chi_{\mathrm{calc}}$) of the interaction parameter between different solvents and the test material [33]. For each solvent, the specific retention volume $V_{g}^{0}$ of the probe to the test material is obtained by the IGC experiment. The interaction parameter $\chi_{12}^{\infty }$ is obtained by Equation (6) and is defined as the experimental value of the interaction parameter ($\chi_{\exp}$). However, the interaction parameters depend on the three HSPs of the solvent probe and the test material, particularly the HSP distance Ra of the two, as defined in Equation (3). From *R*_a_, we derive the calculated value of the interaction parameter ($\chi_{\mathrm{calc}}$):

(10) $$\chi_{\mathrm{calc}}=\frac{R_{a}^{2}.V_{2}}{4RT}$$

where *R* is the universal gas constant, *V*_2_ is the molar volume of the polymer in the stationary phase, *T* is the column temperature, and *R*_a_ is the distance between the solvent probe and the HSPs of the test material, calculated by estimating the HSPs of the test material in advance. The fitting value *R*^2^ between the experimental value ($\chi_{\exp}$) and the calculated value ($\chi_{\mathrm{calc}}$) is optimized and maximized by a large number of estimates of the HSPs of the test material, and the final estimated HSPs are the HSPs of the test material.

#### 2.3.2. IGC Experiment

The inverse gas chromatograph (NeuronIC) was provided by Adscientis, Wittelsheim, France. The data are recorded by the Nucleus software (Adscientis, version 3.3.3). Impregnation of chromatographic support with Nafion was described as follows: 0.1891 g of Nafion was dissolved in ethanol at room temperature. Then, chromatographic support (Chromosorb), with a mass of 0.7833 g after acid washing, was added to this solution for impregnation, resulting in an impregnation ratio of 19.4%. The impregnating solvent was evaporated at 60 °C, and then the Chromosorb impregnated with polymer was introduced into columns with a diameter of 4.5 cm and length of 30 cm. These columns were placed under a flow of helium at 75 °C overnight to remove traces of ethanol (and other VOCs). The measurements were also performed at 75 °C, under a helium flow rate of 20 mL/min. The specific retention volume ($V_{g}^{0}$) and interaction parameters ($\chi_{12}^{\infty }$) were obtained, and the HSPs of Nafion were calculated.

### 2.4. Group Contribution (GC) Method

Early methods to estimate the Hansen solubility parameters were developed for polymers by Hoftyzer–Van Krevelen [34], and for solvents by Hoy [35]. The modern group contribution method used to estimate the solubility parameters of complex organic structures was created by Stefanis–Panayiotou [36,37]; it is second-order group contribution method. Given the presence of the -SO_2_ group in the sulfonic acid group, the HSPs of Nafion in this work were calculated by the Hoftyzer–Van Krevelen and Stefanis–Panayiotou group contribution methods.

The Hoftyzer–Van Krevelen method is one of the most common methods of group calculation, where each parameter can be estimated as follows:

(11) $$\delta_{d}=\frac{\sum F_{\mathrm{di}}}{V}$$

(12) $$\delta_{p}=\frac{\sqrt{\sum F_{\mathrm{pi}}^{2}}}{V}$$

(13) $$\delta_{h}=\sqrt{\frac{\sum E_{\mathrm{hi}}}{V}}$$

where *F*_di_ is the contribution of group *i* to the molar attraction constant dispersion component *F*_d_, and *F*_pi_ is the contribution of group *i* to the polar component *F*_p_. When two identical polar groups are present in the symmetrical position, the value of *δ*_p_, as calculated by Equation (12), is multiplied by the symmetry factor, which is 0.5 for a plane of symmetry [34]. For two planes of symmetry, this value is multiplied by 0.25. For multiple symmetry planes, the value is multiplied by 0. *E*_hi_ is the hydrogen bond energy of each structural group *i*. For molecules with multiple planes of symmetry, *δ*_h_ = 0.

The Stefanis–Panayiotou method is characterized by describing the molecular structure of organic compounds with two functional groups and then dividing the structural units of the compounds into primary groups of a basic molecular structure and secondary groups based on conjugation theory. When *δ*_p_ > 3 MPa^1/2^ and *δ*_h_ > 3 MPa^1/2^, the equation for estimating Hansen solubility parameters is as follows [36]:

(14) $$\delta_{d}={\left(\sum_{i}N_{i}C_{i}+\sum_{j}M_{j}D_{j}+959.11\right)}^{0.4126}{\mathrm{MPa}}^{\left(1/2\right)}$$

(15) $$\delta_{p}=\left(\sum_{i}N_{i}C_{i}+\sum_{j}M_{j}D_{j}+\;7.6134\right){\mathrm{MPa}}^{\left(1/2\right)}$$

(16) $$\delta_{h}=\left(\sum_{i}N_{i}C_{i}+\sum_{j}M_{j}D_{j}+\;7.7003\right){\mathrm{MPa}}^{\left(1/2\right)}$$

where *C_i_* is the contribution of the *i*-type first-order group with *N_i_* occurrences in the compound, and *D_j_* is the contribution of the *j*-type second-order group with *M_j_* occurrences in the compound. For compounds without second-order groups (where $\sum_{j}M_{j}D_{j}$ = 0), in this study on Nafion, since Nafion does not contain secondary groups, only the group contributions of first-order groups were considered.

## 3. Results and Discussion

### 3.1. HSPiP Method

Table 1 summarizes the solubility test results of Nafion in the fifty organic solvents used. The HSPs of these solvents can be found in Hansen et al. [17] (pp. 347–483). The results show that among the fifty organic solvents tested, Nafion can be dissolved in most solvents, which typically exhibit strong hydrogen bond affinity via hydroxyl groups in the side-chain -SO_3_H groups in Nafion, such as ethanol, 1-propanol, acetic acid, and 2-phenoxy ethanol, among others. In addition, some solvents containing polar groups, such as -C=O, also have a strong relationship with Nafion, such as ethyl acetate, cyclohexanone, and diacetone alcohol.

**Figure 2:**
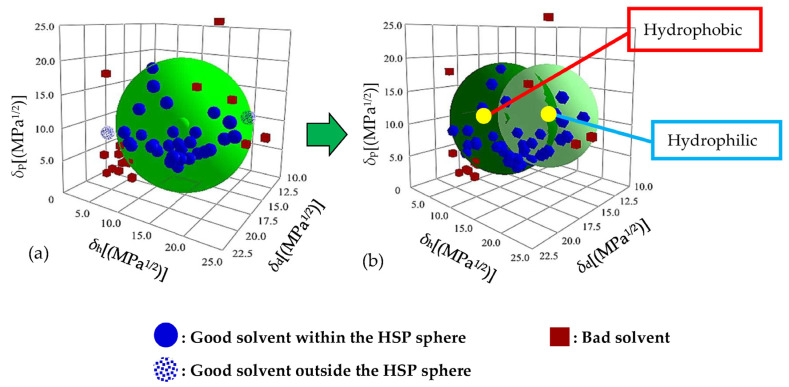
Hansen solubility spheres of Nafion, under the (**a**) “Classic” and (**b**) “Double Sphere” methods.

Based on the solubility test results shown in Table 1, the Hansen solubility spheres of Nafion were fitted with the “Classic” method (single sphere) and the “Double Sphere” method (double sphere) using the HSPiP software, as shown in Figure 2. Figure 2a shows that the interaction radius of the solubility sphere fitted using the classical single-sphere method is large (*R*_o_ = 10.7). The fitting results show abnormal solvent points, i.e., the good solvents, such as acetophenone (smaller *δ*_h_) and methanol (larger *δ*_h_), which are supposed to be inside the sphere, are fitted to the outside of the sphere. This is because *δ*_h_ is associated with the ability to form hydrogen bonds, and the distribution area of good solvent points in the direction of the *δ*_h_ axis is wide. This indicates that the polymer molecule has non-polar and polar parts, which show poor and strong abilities to form hydrogen bonds, respectively. Since the single-sphere method failed to converge within a unified solubility sphere, the Hansen solubility sphere of Nafion was fitted by the double-sphere method, as shown in Figure 2b. The good solvent points fall into the solubility sphere and the fitting accuracy is high, indicating that Nafion has dual solubility parameters. This is because Nafion is similar to a surfactant and has a hydrophobic backbone and a hydrophilic side chain. The hydrophobic backbone (marked in red) is easily soluble in non-polar solvents, such as acetophenone, and does not easily form hydrogen bonds. The hydrophilic side chain (marked in blue), with the sulfonate group, can easily form hydrogen bonds with polar solvents, such as methanol. Surfactants consist of hydrophobic and hydrophilic components, and the calculation of the HSPs generally involves the double-sphere method [29].

The test results obtained by the software show the hydrophilic parts: *δ*_d_ = 15.2 MPa^1/2^, *δ*_p_ = 11.7 MPa^1/2^, *δ*_h_ = 15.9 MPa^1/2^, and *R*_o_ = 7.7; and the hydrophobic parts: *δ*_d_ = 16.4 MPa^1/2^, *δ*_p_ = 10.5 MPa^1/2^, *δ*_h_ = 8.9 MPa^1/2^, and *R*_o_ = 8.8.

### 3.2. IGC Method

The solvent molecules of the IGC experiment were acetone, acetonitrile, diethyl ether, ethanol, methyl acetate, 2-propanol, pyridine, toluene, and o-xylene. According to Equations (5) and (6), $V_{g}^{0}$ and $\chi_{12}^{\infty }$ between Nafion and probe molecules were calculated, and the results are shown in Table 2.

Flory–Huggins interaction parameters (${\;\chi }_{12}^{\;\infty \;})$ are commonly used to evaluate the thermodynamic miscibility of polymer–solvent systems. The larger the ${\;\chi }_{12}^{\;\infty \;}$, the worse the solubility. Generally, at $\chi_{12}^{\infty }$ < 0.5, the polymer can be dissolved in the selected solvent, indicating that the selected solvent is a good solvent. When 0.5 < $\chi_{12}^{\infty }$ < 1, solvent solubility is moderate, corresponding to a medium solvent. At $\chi_{12}^{\infty }$ > 1, the polymer is immiscible with the selected solvent, indicating it is a poor solvent [24].

Table 2 shows that the interaction parameters of toluene and o-xylene are larger than those of other probe solvents. They act as poor solvents for Nafion, and the other probe solvents act as good solvents for Nafion. These observations are consistent with the solubility test results in Table 1. Furthermore, in the IGC experiment on Nafion, the interaction between the probe solvent and Nafion resulted in two extremes. For high-polarity solvents, such as ethanol and 2-propanol, the duration of action of the solvent probe with Nafion was long. However, for non-polar solvents, such as *n*-alkane, isooctane, and cyclooctane, the interaction time between the probe and Nafion was very short. Furthermore, since most non-polar probes could not easily diffuse into the polymer structure, only nine types of solvents (of about twenty types, in general) were found in the IGC results.

**Figure 3:**
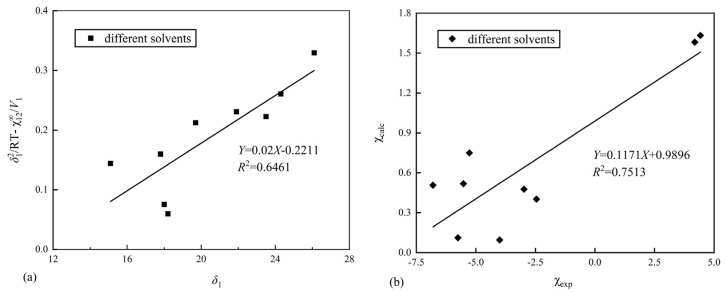
(**a**) Fitting plot of *δ*_1_ and $\delta_{1}^{2}$/*RT*${-\chi }_{12}^{\infty }$/*V*_1_; (**b**) fitting plot of the experimental ($\chi_{\exp}$) and calculated ($\chi_{\mathrm{calc}}$) values.

The interaction parameter ($\chi_{12}^{\infty }$) obtained from Table 2 is taken as the experimental value ($\chi_{\exp}$) of the interaction parameter, and the calculated value ($\chi_{\mathrm{calc}}$) of the interaction parameter is obtained by estimating the HSPs of Nafion. The experimental ($\chi_{\exp}$) and the calculated ($\chi_{\mathrm{calc}}$) values are linearly fitted, and the predicted value of Nafion is continuously optimized, so that the fitting degree *R*^2^ reaches the maximum. The fitting diagram of the final experimental ($\chi_{\exp}$) and calculated ($\chi_{\mathrm{calc}}$) values is shown in Figure 3.

Based on Equation (9), the Hildebrand solubility parameter of Nafion is calculated, as shown in Figure 3a. From the fitted line, it can be seen that the goodness of fit, *R*^2^, is relatively low, indicating a poor fit. The Hildebrand solubility parameter of Nafion, *δ*_2_, is calculated from the slope of the line as 28.9 MPa^1/2^.

The maximum fitting degree of the experimental ($\chi_{\exp}$) and calculated ($\chi_{\mathrm{calc}}$) values is *R*^2^ = 0.7513, as shown in Figure 3. Here, the estimated HSPs of Nafion are *δ*_d_ = 14.2 MPa^1/2^, *δ*_p_ = 8.7 MPa^1/2^, and *δ*_h_ = 9.3 MPa^1/2^.

### 3.3. GC Method

The HSPs of Nafion in this work were calculated by the Hoftyzer–Van Krevelen and Stefanis–Panayiotou group contribution methods.

#### 3.3.1. Hoftyzer–Van Krevelen Group Contribution Method

The Hoftyzer–Van Krevelen group contribution method was used to calculate the HSPs. For polymers, the types and quantities of groups were obtained from their structural units. The structural units of Nafion are shown in Figure 1.

Table 3 summarizes the types and quantities of groups in the Nafion polymer structural units. In Krevelan et al. [34] and Jian et al.’s [38] works, the group contributions *F*_di_, *F*_pi_, and *E*_hi_ of the related structural groups are available. “Total” represents the result when adding the corresponding group contribution value according to the formula $\;\sum F_{\mathrm{di}},\;\sum F_{\mathrm{pi}}^{2},\;\sum E_{\mathrm{hi}}$. Using Equations (11)–(13), the HSPs for Nafion can be calculated as *δ*_d_ = 14.4 MPa^1/2^, *δ*_p_ = 5.8 MPa^1/2^, and *δ*_h_ = 11.4 MPa^1/2^.

#### 3.3.2. Stefanis–Panayiotou Group Contribution Method

The HSPs were also calculated using the Stefanis–Panayiotou group contribution method. The types and quantities of groups in the Nafion polymer structural units are shown in Table 4. According to Panayiotou et al. [36], the group contributions, *δ*_d_, *δ*_p_, and *δ*_h_, of the related structural groups can be obtained. By substituting the data corresponding to each group in Table 4 into the Equations (14)–(16), the HSP values of Nafion can be calculated as *δ*_d_ = 13.5 MPa^1/2^, *δ*_p_ = 24.6 MPa^1/2^, and *δ*_h_ = 13.6 MPa^1/2^.

### 3.4. Comparison and Discussion of the HSPs of Nafion Obtained by Different Methods

The calculation results of HSPs of Nafion in this paper are listed in Table 5. Yeo et al. first studied the Hildebrand parameters of Nafion by the swelling method in 1980 [13]. Dual SPs of the Nafion ionomer were obtained from different solvent swelling experiments, which could be attributed to the hydrophobic backbone (*δ*_backbone_ = 9.7 cal^1/2^cm^−3/2^) and hydrophilic side chain (*δ*_side chain_ = 17.3 cal^1/2^cm^−3/2^). Hiroyuki et al. of Toyota Motor Corporation also calculated the dual HSPs of PFSA ionomers and applied those to the preparation process of the catalyst ink for proton exchange membrane fuel cells [39]. Welch and Wang published HSPs for the Nafion membranes N 115 (membrane thickness: 127 mm) and N 117 (membrane thickness: 183 mm) [40,41], and these results are presented in Table 5 for comparison.

Among the three different HSP methods reported in this paper, the HSPs of Nafion calculated by the IGC method are smaller than those calculated by the other two methods. The possible reason is that some of the gaseous solvent probes may not diffuse into the polymer during the IGC test, which affects the final fitting accuracy. For a flexible polymer with a lower glass transition temperature (*T*_g_ = 100 °C) [42], such as Nafion, its free volume becomes apparent only when the temperature is higher than its *T*_g_. Additionally, the larger the free volume, the faster the diffusion rate. Therefore, it is not advisable to use the IGC method to measure the HSPs of polymers whose test temperature of IGC is lower than its *T*_g_. In the results of the group contribution method, it can be seen that the results obtained by the two group contribution methods are significantly different, especially the *δ*_p_ values. This unreasonable result may arise from not considering the molecular weight of the polymer or its concentration. It should be noted that the dual solubility parameters calculated by the HSPiP method are in good agreement with the dual characteristics of Nafion, with both the hydrophobic backbone and hydrophilic side chain. The Nafion dual SPs obtained by the HSPiP method are very close to those mentioned in the Toyota patent [39]. However, the patent does not provide the source of the HSPs of Nafion. Therefore, we believe that the dual Hansen solubility parameters calculated by the HSPiP method are reasonable. This study focuses on Nafion as the research subject; however, the research method presented here is not limited to Nafion. It is also applicable to fuel cell polymers with different sulfonate side chains, varying side-chain lengths of PFSA, or phosphate side chains. Related studies could be expanded in future research.

## 4. Conclusions

Three different methods were used to calculate the Hansen solubility parameters of Nafion. The results show that the IGC method is suitable for the calculation of HSPs of most substances. However, it is not suitable for Nafion, a polymer with a special structure, because the probe molecule cannot interact with the polymer. This reduces the fitting degree of the test results and affects the accuracy of the results. The HSPs calculated by the different methods in the group contribution method show significant differences. For Nafion copolymerized by the perfluorovinyl ether sulfonyl fluoride and tetrafluoroethylene (TFE), it is crucial to clearly determine the proportions of the two monomers in the polymer. The group contributions of the two monomers were then calculated separately according to the ratio, which may improve the accuracy of the group contribution method. HSPiP software was used to fit the dual HSPs of Nafion. HSPs corresponding to the hydrophobic backbone and hydrophilic side chain of Nafion were *δ*_d_ = 16.4 Mpa^1/2^, *δ*_p_ = 10.5 MPa^1/2^, and *δ*_h_ = 8.9 MPa^1/2^; and *δ*_d_ = 15.2 MPa^1/2^, *δ*_p_ = 11.7 MPa^1/2^, and *δ*_h_ = 15.9 MPa^1/2^, respectively. It is reasonable to calculate the solubility parameters of Nafion using the HSPiP method. The calculation of the HSPs of Nafion provides an effective theoretical basis for the solvent selection of Nafion, and also provides an important parameter for optimizing the formulation and preparation process of the catalyst ink. We will prepare catalyst ink using the method proposed in this paper to select solvents in future work.

## Figures and Tables

**Table 1:** Solubility test results of Nafion and 50 solvents.

Solvent	*δ*_d_ (MPa^1/2^)	*δ*_p_ (MPa^1/2^)	*δ*_h_ (MPa^1/2^)	Solubility Test	RED	V
Formic Acid	14.6	11.9	16.6	1	0.176	37.9
Diacetone Alcohol	15.8	8.2	10.8	1	0.365	124.3
Propylene Glycol Monomethyl Ether	15.6	6.3	11.6	1	0.595	98.2
Ethanol	15.8	8.8	19.4	1	0.603	58.6
Ethylene Glycol Monoethyl Ether	15.9	7.2	14	1	0.661	97.5
1-Hexanol	15.9	5.8	12.5	1	0.683	124.9
1-Propanol	16	6.8	17.4	1	0.693	75.1
Cyclohexanone	17.8	6.3	5.1	1	0.718	104.2
2-Propanol	15.8	6.1	16.4	1	0.744	76.9
Methanol	15.1	12.3	22.3	1	0.824	40.6
Dimethyl Sulfoxide	18.4	16.4	10.2	1	0.828	71.3
1-Pentanol	15.9	4.5	13.9	1	0.896	108.6
Cyclohexanol	17.4	4.1	13.5	1	0.927	106
n-Methyl-2-Pyrrolidone	18	12.3	7.2	2	0.464	96.6
Dimethyl Formamide	17.4	13.7	11.3	2	0.513	77.4
Tetrahydrofuran	16.8	5.7	8	2	0.562	81.7
Propylene Glycol Monomethyl Ether Acetate	15.6	5.6	9.8	2	0.593	137.1
Acetic Acid	14.5	8	13.5	2	0.602	57.1
Ethyl Acetate	15.8	5.3	7.2	2	0.634	98.5
Dibasic Esters	16.2	4.7	8.4	2	0.662	159
Methylene Dichloride	18.2	6.3	6.1	2	0.706	63.9
Pyridine	19	8.8	5.9	2	0.712	80.9
Diethylene Glycol	16.6	12	20.7	2	0.714	95.3
Ethylene Glycol Monobutyl Ether	16	5.1	12.3	2	0.731	131.6
Methyl Isobutyl Ketone	15.3	6.1	4.1	2	0.777	125.8
1,3-Butanediol	16.6	10	21.5	2	0.834	90
m-Cresol	18	5.1	12.9	2	0.85	104.7
Benzyl Alcohol	18.4	6.3	13.7	2	0.86	103.8
t-Butyl Alcohol	15.2	5.1	14.7	2	0.869	95.8
2-Phenoxy Ethanol	17.8	5.7	14.3	2	0.885	124.7
2-Ethyl-Hexanol	15.9	3.3	11.8	2	0.889	156.6
1-Phenyl-1-Butanone	18	5.6	3.6	2	0.897	152.2
Acetonitrile	15.3	18	6.1	2	0.943	52.6
Ethanolamine	17	15.5	21.2	2	0.959	59.8
Acetophenone	19.6	8.6	3.7	2	0.964	117.4
Phenetole	18.4	4.5	4	2	0.992	127.2
Propylene Glycol	16.8	9.4	23.3	3	1.079	73.7
1,4-Dioxane	19	1.8	7.4	3	1.167	85.7
Ethylene Glycol	17	11	26	3	1.383	55.9
Propylene Carbonate	20	18	4.1	4	1.305	85
Trichloroethylene	18	3.1	5.3	5	1.004	90.1
Chlorobenzene	19	4.3	2	5	1.209	102.1
o-Xylene	17.8	1	3.1	5	1.304	121.1
Carbon Tetrachloride	17.8	0	0.6	5	1.553	97.1
Heptane	15.3	0	0	5	1.582	147
Water	18.1	17.1	16.9	6	1.033	18
Cyclohexane	16.8	0	0.2	6	1.551	108.7
Hexane	14.9	0	0	6	1.598	131.4
Pentane	14.5	0	0	6	1.62	116
Formamide	17.2	26.2	19	6	1.984	39.9

**Table 2:** Molar volume (*V*_1_), solubility parameters (*δ*_1_), specific retention volume ($V_{g}^{0}$), and interaction parameters ($\chi_{12}^{\infty }$) of probe molecules at 75 °C.

Probe Solvent	*V*_1_/(cm^3^/mol)	*δ*_1_/(J/cm^3^)^1/2^	$V_{g}^{0}/(mL/g)$	$\chi_{12}^{\infty }$
Acetone	73.8	19.7	15,059.04	−5.75
Acetonitrile	52.9	24.3	2782.91	−2.98
Diethyl Ether	104.7	15.1	17,398.04	−6.80
Ethanol	58.6	26.1	30,232.27	−5.52
Methyl Acetate	79.8	17.8	2491.31	−4.00
2-Propanol	76.9	23.5	2455.59	−2.46
Pyridine	80.9	21.9	58,667.74	−5.27
Toluene	106.6	18.2	2.98	4.19
o-Xylene	121.1	18	6.71	4.43

**Table 3:** Calculation of Hoftyzer–Van Krevelen group contribution of Nafion polymer.

Group	Number	*F*_di_ (J/cm^3^)^1/2^mol^−1^	*F*_pi_ (J/cm^3^)^1/2^mol^−1^	*E*_hi_ J/mol	*V*_i_ cm^3^/mol
>C<	9	−70	0	0	3.56
-O-	2	100	400	3000	10
-F-	17	220	0	0	11.2
-SO_2_-	1	590	1460	11,300	32.5
-OH	1	210	500	20,000	9.7
Total	30	4110	2,701,600	37,300	284.64

**Table 4:** Calculation of the Stefanis–Panayiotou group contribution of Nafion polymer.

Group	Number	*δ*_d_	*δ*_p_	*δ*_h_
>SO_2_	1	182.83	11.0254	−0.3602
>O	2	18.09	3.5248	0.0883
>CF-	2	20.32	-	-
-CF_2_-	6	−103.83	-	-
-CF_3_	1	−13.79	−1.9735	−1.2997
-OH	1	−29.97	1.0587	7.3609

**Table 5:** HSPs of Nafion obtained by different methods in this study and HSPs of Nafion in other studies.

Method		*δ*_d_	*δ*_p_	*δ*_h_	Source
HSPiP software	Hydrophilic side chains	15.2	10.7	15.9	This paper
	Hydrophobic backbone	16.4	10.5	8.9	
IGC		14.2	8.7	9.3	
Group contribution	Hoftyzer–Van Krevelen	14.4	5.8	11.4	
	Stefanis–Panayiotou	13.5	24.6	13.6	
Nafion Dispersion, Hiroyuki [39]	Hydrophilic side chains	15.2	11.9	15.9	Other studies
	Hydrophobic backbone	17.7	11.4	8.5	
Nafion N115, Welch [40]		17.4	12.5	9.6	
Nafion N117, Wang [41]		15.1	8.9	9.4	

## Data Availability

The original contributions presented in this study are included in the article. Further inquiries can be directed to the corresponding author.

## Supplementary Materials

The following supporting information can be downloaded at https://www.mdpi.com/article/10.3390/polym17070840/s1, Figure S1: Examples of dissolution and swelling at different scales (scale 1–6).
