# Supplementary material for: Study on the Calculation Method of Hansen Solubility Parameters of Fuel Cell Ionomers
Source: Polymers (Basel). 2025 Mar 21;17(7):840. doi: 10.3390/polym17070840 (PMC11991492; doi:10.3390/polym17070840)
Supplement: Supplementary file 1 [file polymers-17-00840-s001.zip › polymers-3471935-supplementary.pdf]

# Supporting Informat

# Study on the Calculation Method of Hansen Solubility Parameters of Fuel Cell Ionomers

**Chao Meng [1,2,3], Shang Li [1,2,3,*], Qianyun Wu [1,3], Mengyu Li [1,3], Shenao Tian [1,2,3], Haolin Tang [1,2,3] and Mu Pan [1,2,3]**

[1] State Key Laboratory of Advanced Technology for Materials Synthesis and Processing,Wuhan University of Technology, Luoshi Road 122#, Wuhan 430070, China; 331045@whut.edu.cn (C.M.)

[2] National Energy Key Laboratory for New Hydrogen-Ammonia Energy Technologies, Foshan Xianhu Laboratory, No. 1 Yangming Road, Danzao Town, Nanhai District, Foshan 528200, China

[3] Hubei Key Laboratory of Fuel Cells, Wuhan University of Technology, Wuhan 430070, China

* Correspondence: lishang@whut.edu.cn

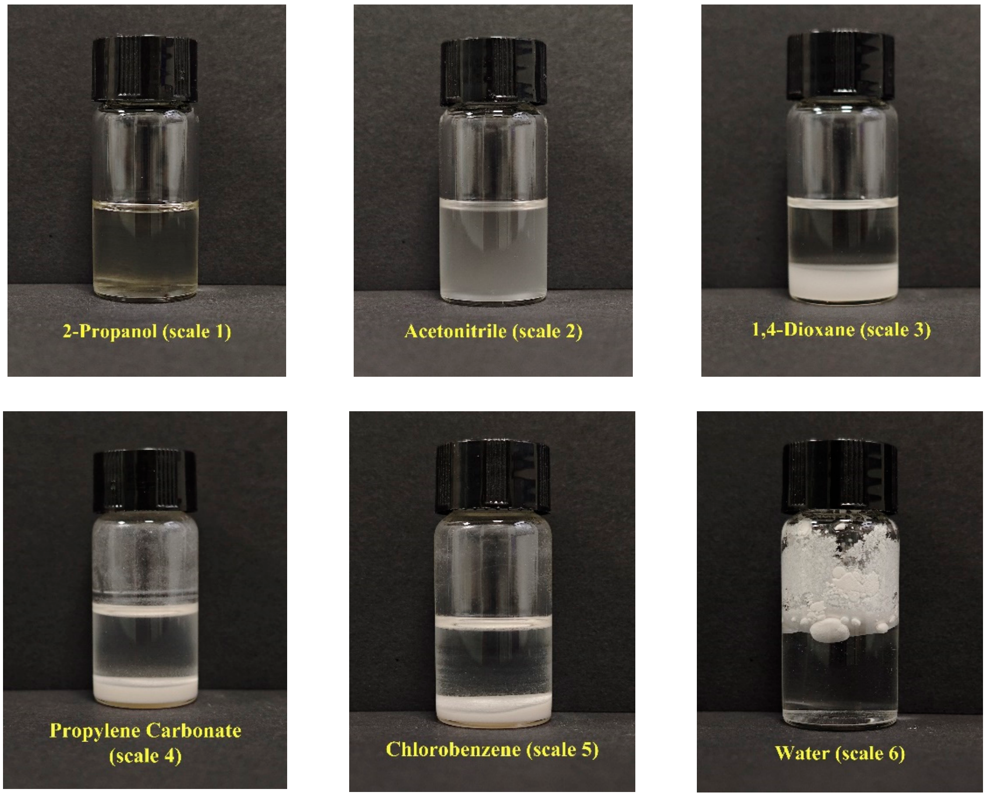


**Figure S1.** Examples of dissolution and swelling at different scales (scale 1-6).
